# The diagnostic accuracy of point-of-care nucleic acid-based isothermal amplification assays for scrub typhus: a systematic review and meta-analysis

**DOI:** 10.3389/fmicb.2024.1516921

**Published:** 2025-01-06

**Authors:** Rashi Dixit, Sandeep Manikandan, John Antony Jude Prakash, Manisha Biswal, Dharitri Mohapatra, Natarajan Gopalan, G. Gnanamani, Sujit Kumar Behera

**Affiliations:** ^1^Department of Epidemiology & Public Health, Central University of Tamil Nadu, Thiruvarur, India; ^2^Christian Medical College, Vellore, India; ^3^Post-graduate Institute of Medical Education and Research, Chandigarh, India; ^4^S.C.B. Medical College and Hospital, Cuttack, India

**Keywords:** loop-mediated isothermal amplification, diagnostics, scrub typhus, meta-analysis, *Orientia*

## Abstract

**Introduction:**

The diagnosis and detection of pathogens such as *Rickettsia* and *Orientia* is a cause of major concern among the public health community. Unavailability of rapid, cost-effective diagnostic assays contributes to delayed diagnosis and timely treatment. Using the methodology of systematic reviewing and meta-analysis, the study aimed to synthesize and compare the diagnostic performances of all the available isothermal assays for the detection of classical rickettsial diseases.

**Methods:**

Studies were retrieved from PubMed and Scopus, and selection and screening were conducted using pre-determined inclusion and exclusion criteria. Analysis was performed using Meta-DiSc 2.0 for the assessment and comparison of diagnostic performance of the isothermal assays.

**Results:**

Overall, six studies were selected as a part of this systematic review. All the selected studies (*n* = 6) optimized LAMP as their index test to detect scrub typhus. The quality assessment of the selected studies revealed only (*n* = 1) study to be of poor quality with a QUADAS-2 score of (<2). Meta-analysis revealed the pooled sensitivity of LAMP to be 66% [95% CI (0.40–0.85)] with a pooled specificity of 94% [95% CI (0.81–0.98)]. LAMP was estimated with a positive likelihood ratio of 8.3 [95% CI (3.8–18.1)] and a negative likelihood ratio of 0.3 [95% CI (0.2–0.7)] with a false positivity rate of 0.07 [95% CI (0.02–0.2)]. The diagnostic odds ratio was reported to be 21.96 [95% CI (10.2–47.3)]. Due to severe heterogeneity in the body of evidence (*I*^2^ = 0.77), a meta-regression was performed with certain covariates to explore the potential causes. A case–control design was found to exaggerate the sensitivity {0.84 [95% CI (0.5–0.9)]} and specificity {0.73 [95% CI (0.6–0.8)]}.

**Conclusion:**

The findings reveal subpar performance of LAMP for the detection of scrub typhus. Active research and development focused on optimization of novel molecular diagnosis that are efficient, rapid, and cost-effective shall foster timely diagnosis and aid in reduction of the overall burden of scrub typhus.

**Protocol and registration:**

A detailed protocol of this review is registered and available in Prospero at: https://www.crd.york.ac.uk/prospero/. (registration number CRD42024511706).

## 1 Introduction

Members of the family “*Rickettsiaceae*” are the largest contributors to the burden of rickettsioses worldwide and are known to cause classical rickettsial diseases belonging to three major categories. Interestingly, the pathogenic species of *Rickettsia* (31 species) (Abdad et al., 2017; Abdad et al., 2018) were originally classified into the spotted fever group (SFG) or the typhus group (TG) (Abdad et al., 2018; Fang et al., 2017). The SFG *Rickettsia,* notably *R. rickettsii* and *R. conorii,* are known to cause Rocky mountain spotted fever and Mediterranean spotted fever in many parts of America, Europe, Africa, and Asia (Kato et al., 2013; Álvarez-López et al., 2021; Nanayakkara et al., 2013). The TG *Rickettsia* comprising of only two pathogenic species, namely, *R. typhi* and *R. prowazekii,* well known to cause murine typhus and epidemic typhus are transmitted by the infective bite of fleas and lice, respectively (Fang et al., 2017; Rauch et al., 2018). The TG initially also harbored the mite borne pathogen *Orientia tsutsugamushi* primarily called *Rickettsia tsutsugamushi*; however, with the development of novel molecular phylogenetics, the species was re-classified into a novel group, namely, the scrub typhus group (STG) (Abdad et al., 2018; Rauch et al., 2018). Recently, *O. chuto*, a new infectious species of *Orientia,* isolated from an Australian resident who contracted scrub typhus while traveling to Dubai (Izzard et al., 2010), has led to its addition in the STG. The “tsutsugamushi triangle” extending across significant areas of Southeast Asia is endemic to scrub typhus and perhaps likely the most significant rickettsial infection around the globe in terms of disease burden (Salje et al., 2021). Approximately, 1 billion people are thought to be at risk (Paris et al., 2013; Watt and Parola, 2003).

Classical rickettsial infections known to transmit by the infective bite of hematophagous arthropods such as mites, ticks, and fleas (Chugh et al., 2022) mostly manifest within the spectrum of acute febrile illness similar to dengue, chikungunya, and malaria (Kala et al., 2020; Koraluru et al., 2015). This constitutes as a huge challenge to the physicians and indirectly contributes to significant morbidity and mortality. Immunofluorescence assay (IFA) is the preferred gold standard for the diagnosis of *Rickettsia* (Stewart and Stewart, 2021); however, IgM ELISA and PCR serve as the most rapid and cost-effective diagnostics to detect other Rickettsial infections (Abdad et al., 2018; Stewart and Stewart, 2021; Luce-Fedrow et al., 2015). A recently proposed reference standard for the diagnosis of scrub typhus is the scrub typhus infection criteria (STIC), which use a combination of culture-based detection, PCRs, IFA, and ELISA (Paris et al., 2011; Blacksell et al., 2012). A positive STIC is inferred when either (a) *O. tsutsugamushi* is isolated on cell culture and or (b) at least two out of the nested 56 kDa PCR assay, 47 kDa based real-time PCR assay, or groEL-based real-time PCR assay turns positive and or (c) there is an admission IgM titer of ≥1:12,800 or (d) there is at least a 4-fold rise in IFA IgM titer in paired sera samples (Paris et al., 2011; Blacksell et al., 2012). However, application of these routine diagnostic assays requires expensive equipment, skilled manpower, and disposal of extensive resources, a major drawback when testing patient samples in rural, resource poor, and field-based settings.

Isothermal assays offer an alternative to classical diagnostic techniques; they are quick, inexpensive, and highly adaptable to point-of-care (PoC) settings (Dixit et al., 2023). Popular isothermal assays such as loop-mediated isothermal amplification (LAMP), recombinase polymerase amplification (RPA), and closed dumbbell-mediated isothermal amplification (CDA) are widely developed to screen rickettsial agents such as *R. typhi*, *R. prowazekii*, *R. raoultii,* and *R. tarasevichiae* (Xue et al., 2022; Gui et al., 2022; Pan et al., 2012). RPA and LAMP have also been optimized to detect infections caused by *O. tsutsugamushi* (Paris et al., 2008; Chao et al., 2015). Suggestively, literature regarding diagnostic test accuracy of isothermal assays is sparse, highlighting the need for a systematic review on estimating the diagnostic performance and comparative efficiency of the available isothermal assays for the detection of classical rickettsial infections. The current study aims to bridge this gap and, present findings that may aid in the assessment of the currently available isothermal assays and enhance research and development in the field of medical diagnostics.

## 2 Methods

### 2.1 Eligibility criteria

The selection of the articles were determined on the basis of an inclusion and exclusion criteria set in accordance with the research question during registration of the protocol. Studies that focused to assess the diagnostic performance of an isothermal assay (index test) compared to a standard reference test (STICS criteria and or IFA) for rickettsial diseases (spotted fever group, typhus group, and scrub typhus group) were included. Studies with a clinical focus, of sound, and clear methodology with appropriate validation of the assay were selected. To be included in the review, a study should at the least quantify >50 clinical samples/field isolates from an ambulatory healthcare setting utilizing a cross-sectional framework. A study was deemed disqualified on the grounds of absence of sound methodology and inappropriate reference standard to quantify the performance of the test. Only original research articles were selected; review articles, commentaries, or cohort studies with the absence of statistical validation of the test were not considered in the screening phase of the systematic review.

### 2.2 Data sources

To enhance maximum inclusivity, studies were extracted systematically from Scopus and PubMed using pre-determined standard keywords. All the searches were performed on a single day (23 February 2024) to avoid discrepancies related to data retrieval, and our results reflect a compiled systematic analysis of studies published as of 23 February 2024. No studies were retrieved from registers or any other relevant repositories.

### 2.3 Search strategy

A pilot search was performed prior to formal retrieval of studies to get an overall estimate in terms of practicality of the research question. A specific search strategy was applied with pre-determined standard keywords to maintain uniformity in the searches across databases. The following search strategy was utilized to sought relevant literature ([“isothermal amplification” OR “isothermal amplification assay”] AND [“scrub typhus”]; [“isothermal amplification” OR “isothermal amplification assay”] AND [“rickettsia”]). In Scopus, however, the keyword search was limited to “title” and “abstract.” Bibliometric mining was adopted as a supplementary retrieval strategy to enhance inclusion of important and relevant studies that otherwise do not show up in the search scheme due to algorithm issues related to keywords.

### 2.4 Study selection process

All the retrieved searches from the databases were downloaded as .csv files and were converted to excel workbook (.xlx) for screening purposes. Data cleaning involved elimination of the duplicates and compiling all the studies into a separate worksheet. The studies were then retrieved in full text and screened manually by two independent reviewers. A study would be considered a part of the review when selected by both the reviewers; incase a study posed doubts with regards to selection, a third reviewer was consulted and their verdict was considered for the final selection. Studies deemed irrelevant or inconsistent with the research question were excluded from full-text access.

### 2.5 Data collection process and data items

The selected studies underwent data extraction independently by two reviewers. Important descriptive characters and information from each study were extracted and entered into excel worksheets in various columns such as “study,” “year,” “disease agent,” “country,” “sample size,” “index test,” and “reference test” which were compared across the selected studies. Significant diagnostic variables such as “sensitivity,” “specificity,” “positive predictive value,” “negative predictive value,” and “limit of detection” were also documented which would be further required to perform meta-analysis. In case where any of the above diagnostic variables were not reported in the study, raw values from the 2 by 2 contingency tables were computed to calculate the final estimate. A separate worksheet was also developed with 2 by 2 contingency raw values from the diagnostic indicators to be used as a part of the meta-analysis.

### 2.6 Risk of bias and quality assessment

Risk of bias and quality assessment were performed to assess concerns posed by each study with regards to quality and rigor in terms of methods, analysis, and statistical estimates. QUADAS-2 toolkit was followed to assess risk of bias as well as applicability concerns for each study selected in the review. Each study was judged in terms of four critical domains: “patient selection,” “index test,” “reference test,” and “flow and timing” according to certain signaling questions in each of these categories. For risk of bias assessment, a study that posed lower risk for each domain was allotted a score of 1; studies with high risk in a particular domain were awarded 0 score. All studies were scored out of 4; a study with a total score of <3 was considered of “poor quality,” and studies with a score of 3 or more were considered of “good quality.” For applicability assessment, a similar scoring system was followed and studies were assessed for applicability under three categories, namely, “patient selection,” “index test,” and “reference test,” and were scored out of 3 points in total. Studies with low applicability scores (<3) were disqualified from the meta-analysis. Figures were constructed in accordance with the QUADAS-2 template, in order to comprehensively report the findings of bias and quality assessment.

### 2.7 Effect measures

The descriptive characteristics were compared across studies to estimate and draw valid conclusions as a part of the narrative synthesis. Epidemiological diagnostic indicators such as sensitivity, specificity, positive predictive value, negative predictive value, and limit of detection (LOD) were retrieved from each study to assess the performance of the isothermal assays in the meta-analysis.

### 2.8 Methods of data synthesis

The results from the systematic review were mainly synthesized using two strategies: qualitative narrative synthesis and quantitative meta-analysis. From the reported sensitivity and specificity, 2 × 2 contingency values were retrieved from each study for determining the pooled effect estimates. Descriptive characters were compared across studies to critique and comment on the qualitative aspects; a formal meta-analysis was performed to estimate pooled sensitivity, pooled specificity, and an SROC curve statistic. Forest plot were constructed, and a random effects model was applied to eliminate any heterogeneity, encountered during the analysis. An SROC curve was plotted from the sensitivity and specificity values reported in each study to determine the diagnostic test accuracy. In addition, diagnostic odds ratio (DOR) was also estimated for each study with the extracted 2 × 2 contingency table values. The meta-analysis was performed using a freely available web-based software called Meta-DiSc 2.0.^1^ Detailed protocol for this systematic review has been registered in International Prospective Register for Systematic Review (PROSPERO) with registration number CRD42024511706.

## 3 Results

### 3.1 Study selection

**Figure 1:**
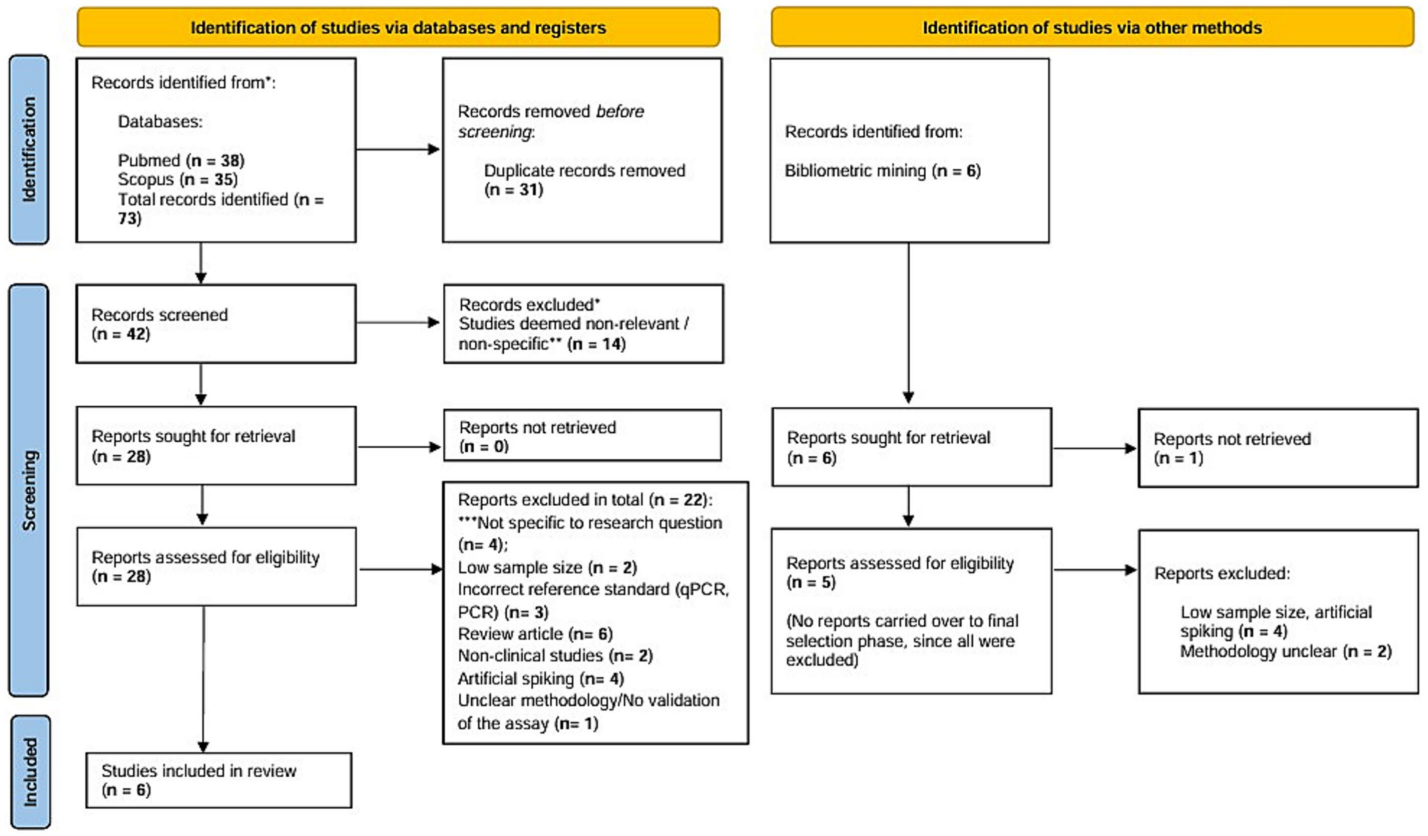
PRISMA flowchart depicting screening and selection of studies for the systematic review and meta-analysis in accordance with PRISMA 2020 checklist. The PRISMA 2020 figure is a template provided at (https://www.prisma-statement.org/prisma-2020-flow-diagram) to be used by researchers conducting Systematic reviews. It is publicly available and is an essential part of reporting standards for systematic review and meta-analysis.

A total of 73 articles were identified through preliminary database searching on PubMed and Scopus, of which 28 studies were retrieved in full text to assess for eligibility against the inclusion criteria. Studies non-specific to the research question (*n* = 14) and duplicates (*n* = 31) were eliminated, and a total of 22 studies were disqualified due to low sample size (*n* = 2), artificial spiking (*n* = 4), usage of reference test other than gold standard (*n* = 3), review articles (*n* = 6), non-clinical studies (*n* = 2), unclear methodology or improper validation of the assay (*n* = 1), and non-specificity to the research question (*n* = 4). Parallelly, bibliometric mining yielded six studies in addition to the traditional database searching, of which none were selected for the final round of review. A total of 28 studies underwent final screening and selection, of which 6 studies were included as a part of this systematic review and meta-analysis. A detailed figure pertaining to the selection and screening phase of the review is pictorially depicted in accordance with Preferred Reporting Items for Systematic Reviews and Meta-Analyses (PRISMA) 2020 flowchart (Figure 1).

### 3.2 Study characteristics

All the selected studies (*n* = 6) optimized isothermal assays to detect *Orientia tsutsugamushi*, the causative organism of scrub typhus. Unfortunately, none of the studies were selected for the detection of Rickettsial diseases due to usage of non-standard reference test in these studies and hence were disqualified during the screening phase. All the studies employed LAMP as their primal index test against different reference standards such as nested PCR (*n* = 1), IFA (*n* = 2), STICS criteria (*n* = 2), and PCR with ELISA (*n* = 1). Two of the studies were affiliated to Thailand, while four studies were optimized in India. Average sample size across these six studies was 206. A brief summary of the descriptive characteristics of each study can be visualized in Table 1.

**Table 1:** Summary of the selected studies and their descriptive characteristics.

Study	Year	Disease agent	Disease outcome	Country	Sample size	Index test	Reference test
Anitharaj et al. (2023)	2023	*Orientia tsutsugamushi*	Scrub typhus	India	280	LAMP	n-PCR
**Paris et al.** **(2011)**	2011	*Orientia tsutsugamushi*	Scrub typhus	Thailand	161	LAMP	STICS
**Karthikeyan et al.** **(2019)**	2019	*Orientia tsutsugamushi*	Scrub typhus	India	50	LAMP	PCR + ELISA
**Roy et al.** **(2021)**	2021	*Orientia tsutsugamushi*	Scrub typhus	India	274	LAMP	IFA
**Kannan et al.** **(2020)**	2020	*Orientia tsutsugamushi*	Scrub typhus	India	316	LAMP	IFA
**Blacksell et al.** **(2012)**	2012	*Orientia tsutsugamushi*	Scrub typhus	Thailand	160	LAMP	STICS

Studies highlighted in bold indicate QUADAS-2 score of > 3).

### 3.3 Risk of bias and quality assessment

**Figure 2:**
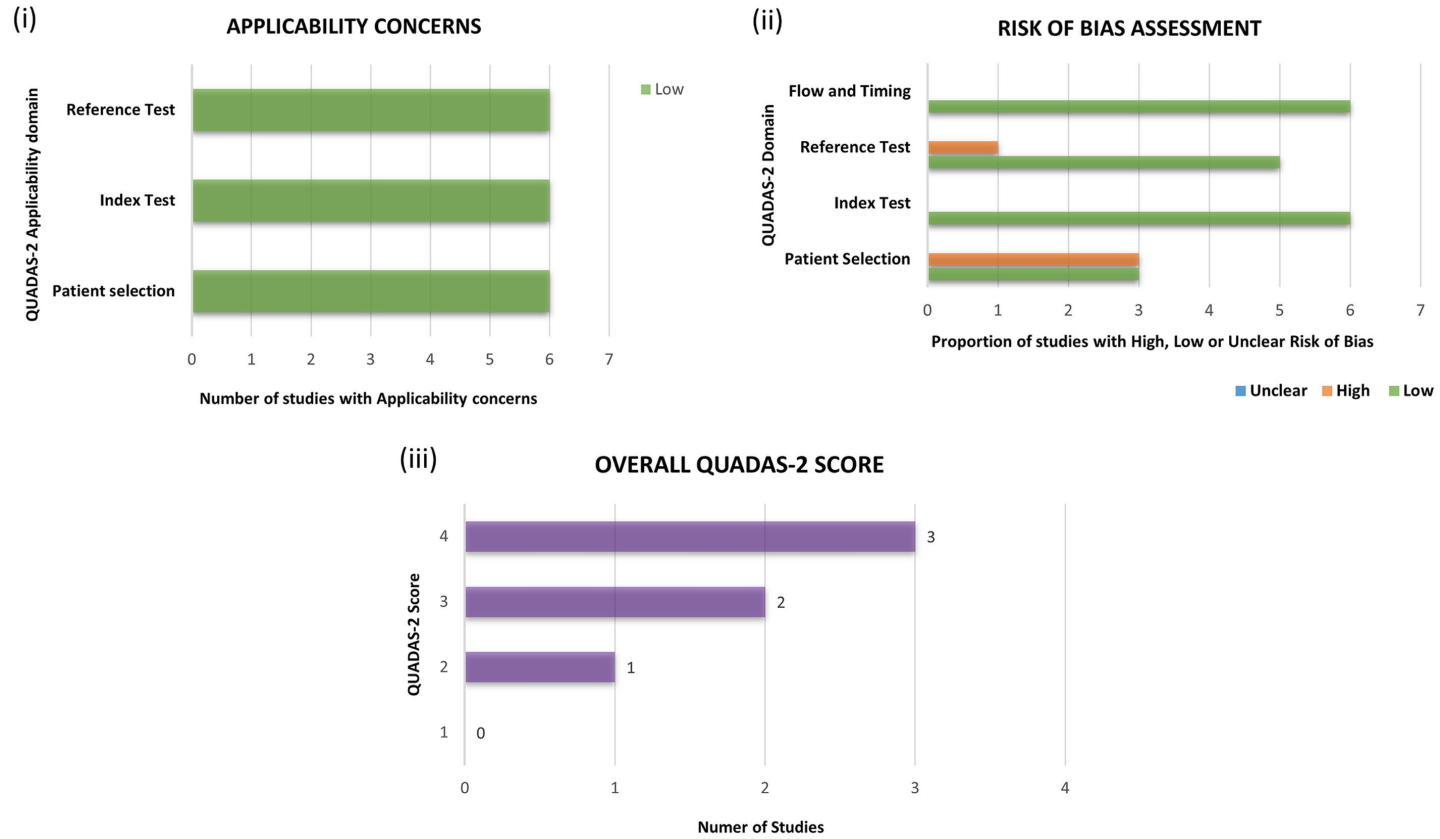
Assessment of the selected studies for quality and risk of bias using the quality assessment of diagnostic accuracy studies-2 (QUADAS-2) toolkit. Each study was assessed for (i) applicability and (ii) risk of bias and, allotted scores in every domain with a final score are depicted in (iii) overall QUADAS-2 score.

Using the standardized QUADAS-2 tool, the reports were assessed for their quality and the potential risk of bias. All the selected studies successfully met the applicability criteria (*n* = 6) and posed no relevant concerns. In terms of risk of bias assessment, three studies were identified posing potential concerns regarding “patient selection” and one study regarding “reference test.” None of the selected reports showed any significant concerns regarding “flow and timing” and usage of appropriate “index test.” Majority of the selected studies (n = 5) were of “good quality” with a QUADAS-2 score of >3. However, one study was deemed to be of “poor quality” with a QUADAS-2 score of 2. A detailed summary and a pictorial description regarding risk of bias and quality assessment can be accessed in Table 2 and Figure 2.

**Table 2:** Assessment of risk of bias and quality concerns of the selected studies using QUADAS-2 (QUADAS-2: quality assessment of diagnostic accuracy studies-2) tool.

Study name	Patient selection	Index test	Reference test	Flow and timing	Quadas-2 score
Anitharaj et al. (2023)	High	Low	High	Low	2
Paris et al. (2011)	Low	Low	Low	Low	4
Karthikeyan et al. (2019)	High	Low	Low	Low	3
Roy et al. (2021)	Low	Low	Low	Low	4
Kannan et al. (2020)	High	Low	Low	Low	3
Blacksell et al. (2012)	Low	Low	Low	Low	4

### 3.4 Performance of the isothermal assays

A compiled detailed summary of epidemiological and diagnostic parameters reported in each of the selected studies is depicted in Table 3. All the included studies had quantified the performance of LAMP against different reference standards (STICS, PCR, ELISA, and IFA) with clinical samples for the detection of *Orientia tsutsugamushi*. The sensitivity of LAMP was found to range from 16% to ~91%, and specificity was found to vary between 70 and 100%. None of the studies reported the limit of detection (LOD) either of the index test or the reference standard.

**Table 3:** Comparative summary of diagnostic and epidemiological parameters as described in each of the selected study.

			Index test					Reference test				
Disease category	Study	Year	SN	SP	PPV	NPV	LOD	SN	SP	PPV	NPV	LOD
Scrub typhus	Anitharaj et al. (2023)	2023	74	70.6	32.3	93.2	*	*	*	*	*	*
Scrub typhus	Paris et al. (2011)	2011	53	94	83	79	*	*	*	*	*	*
Scrub typhus	Karthikeyan et al. (2019)	2019	89	100	100	72	*	*	*	*	*	*
Scrub typhus	Roy et al. (2021)	2021	16.22	99	75	88	*	*	*	*	*	*
Scrub typhus	Kannan et al. (2020)	2020	91	77	80	90	*	95	74	78	94	NA
Scrub typhus	Blacksell et al. (2012)	2012	52.9	94.3	82.6	79.4	*	*	*	*	*	*

*Values not provided.

### 3.5 Meta-analysis

**Figure 3:**
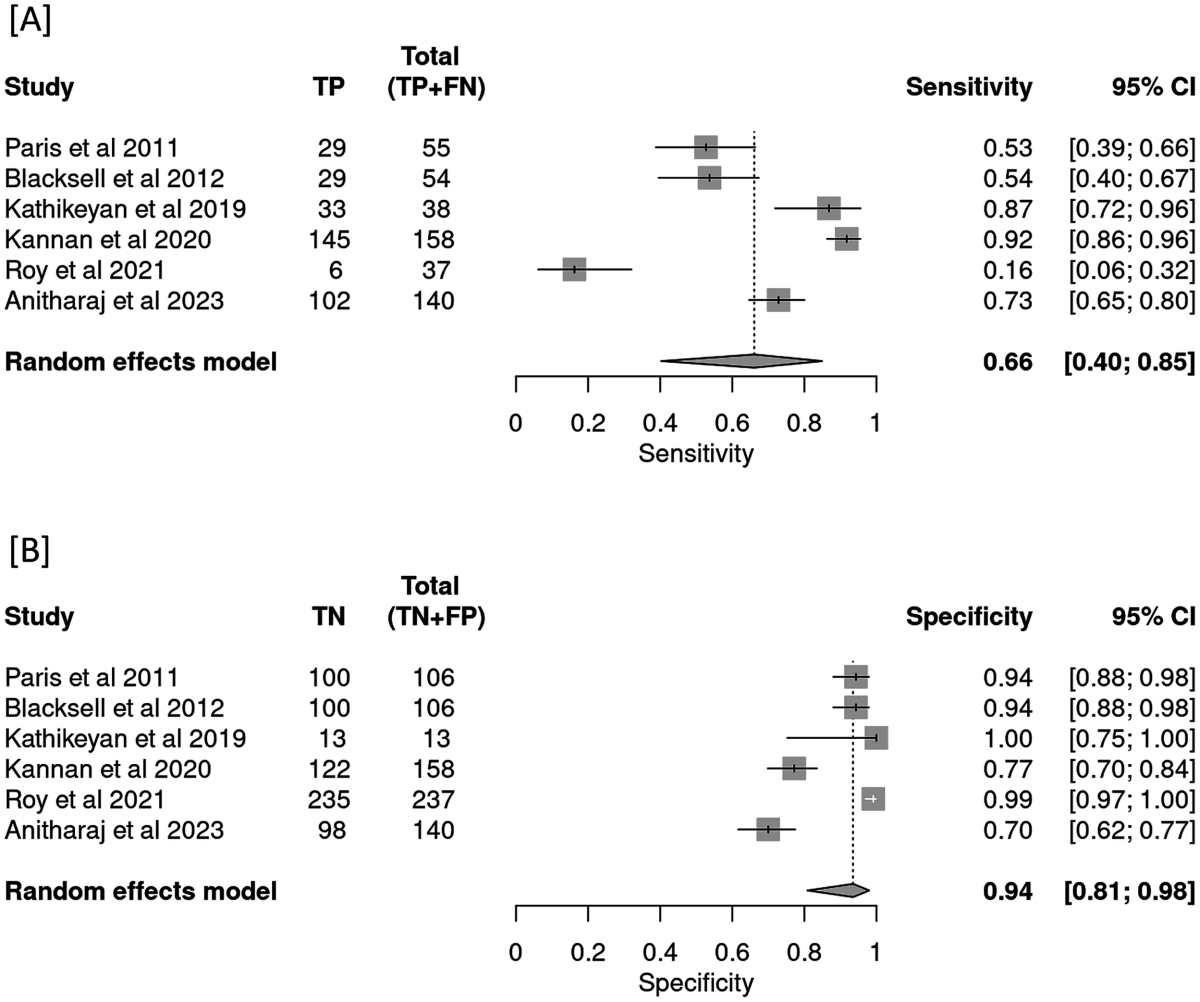
Forest plot depicting pooled effect estimates of **(A)** sensitivity and **(B)** specificity in the meta-analysis.

**Figure 4:**
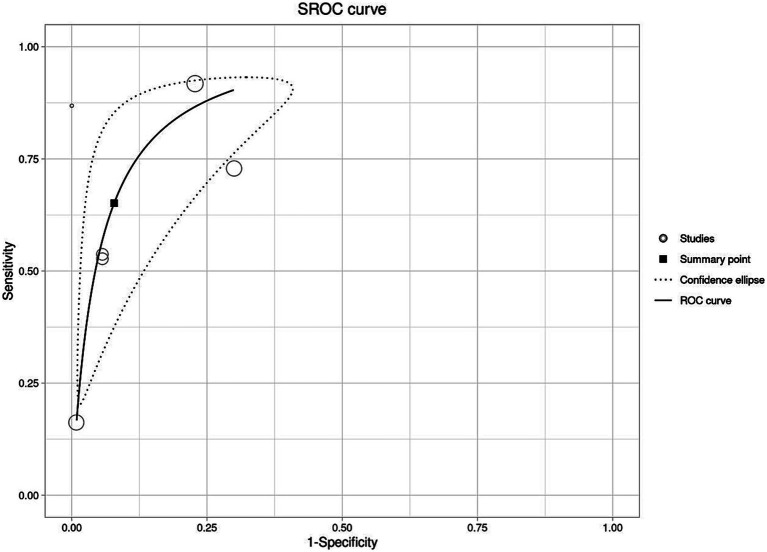
Graphical representation of the diagnostic performance of LAMP as an isothermal assay for the detection of scrub typhus using summary receiver operating curve (SROC).

All the six studies were included in the meta-analysis irrespective of their QUADAS-2 score or quality. Forests plot in Figure 3 depicts pooled effect estimates of sensitivity (Figure 3A) and specificity (Figure 3B). Applying the random effects model, pooled sensitivity of LAMP is reported to be 66% [95% CI (0.40–0.85)], and pooled specificity is reported to be 94% [95% CI (0.81–0.98)]. In the bivariate analysis (Table 4), the index test is estimated with a positive likelihood ratio of 8.3 [95% CI (3.8–18.1)] and a negative likelihood ratio of 0.3 [95% CI (0.2–0.7)] with a false positivity rate of 0.07 [95% CI (0.02–0.2)]. The diagnostic odds ratio of LAMP was estimated to be 21.96 [95% CI (10.2–47.3)]. An SROC curve summarizing the diagnostic performance and the pooled effect size can be visualized in Figure 4 (see web only Supplementary File S1 which includes a 2 by 2 contingency table utilized in the meta-analysis including results of the univariate analysis).

**Table 4:** Glimpse of summary statistics and heterogeneity analysis in the bivariate model of the meta-analysis.

Summary statistics				Heterogeneity analysis	
	Estimate	95% LCI	95% UCI		Estimate
Sensitivity	0.651	0.395	0.842	Var logit (sen)	1.612
Specificity	0.922	0.796	0.973	Var logit (spe)	1.617
DOR	21.966	10.2	47.303	MOR sensitivity	3.357
LR+	8.315	3.805	18.174	MOR specificity	3.363
LR–	0.379	0.203	0.704	Bivariate I^2^	0.771
FPR	0.078	0.027	0.204	Area 95% Prediction Ellipse	0.46

The bivariate analysis also reported significant heterogeneity in the body of evidence (*I*^2^ = 0.77). To decipher the nature of the heterogeneity in this analysis, a meta-regression was pursued on different existing covariates to find a plausible explanation. No significant heterogeneity was encountered as a result of QUADAS-2 score or usage of different reference standards. However, heterogeneity due to employment of different study designs (cross-sectional or case–control) was found to be significant (*p* = 0.005) (see Table 5). Moreover, a cross-sectional study design reportedly undermines the pooled effect estimates {sensitivity—0.52 [95% CI; (0.2–0.7)]; specificity—0.96 [95% CI (0.93–0.98)]}, and a case–control design was found to exaggerate the sensitivity {0.84 [95% CI (0.5–0.9)]} and specificity {0.73 [95% CI (0.6–0.8)]}. In addition, sensitivity analysis (see Table 6) was performed to understand the change in effect estimates due to outlier values. The sensitivity changed to 0.59 [95% CI; 0.31–0.82], and specificity was reported to be 0.92 [95% CI (0.77–0.97)].

**Table 5:** Compiled summary statistics using ‘study design’ as a covariate in the bivariate model of the meta-regression.

Parameter	Estimate case–control	95% LCI case–control	95% UCI case–control	Estimate cross-sectional	95% LCI cross-sectional	95% UCI cross-sectional
Sensitivity	0.845	0.559	0.959	0.526	0.277	0.763
Specificity	0.739	0.61	0.836	0.966	0.934	0.983
DOR	15.376	4.287	55.145	31.577	10.506	94.906
LR+	3.233	2.206	4.737	15.489	7.498	31.995
LR-	0.21	0.066	0.673	0.491	0.282	0.853
FPR	0.261	0.164	0.39	0.034	0.017	0.066

**Table 6:** Summary statistics from the sensitivity analysis.

	Estimate	95% LCI	95% UCI
Sensitivity	0.598	0.318	0.826
Specificity	0.922	0.773	0.976
DOR	17.545	8.945	34.414
LR+	7.652	3.471	16.87
LR-	0.436	0.236	0.805
FPR	0.078	0.024	0.227

Results indicate pooled effect estimates from the meta-analysis after elimination of an outlier value (a study with low sample size).

**Figure 5:**
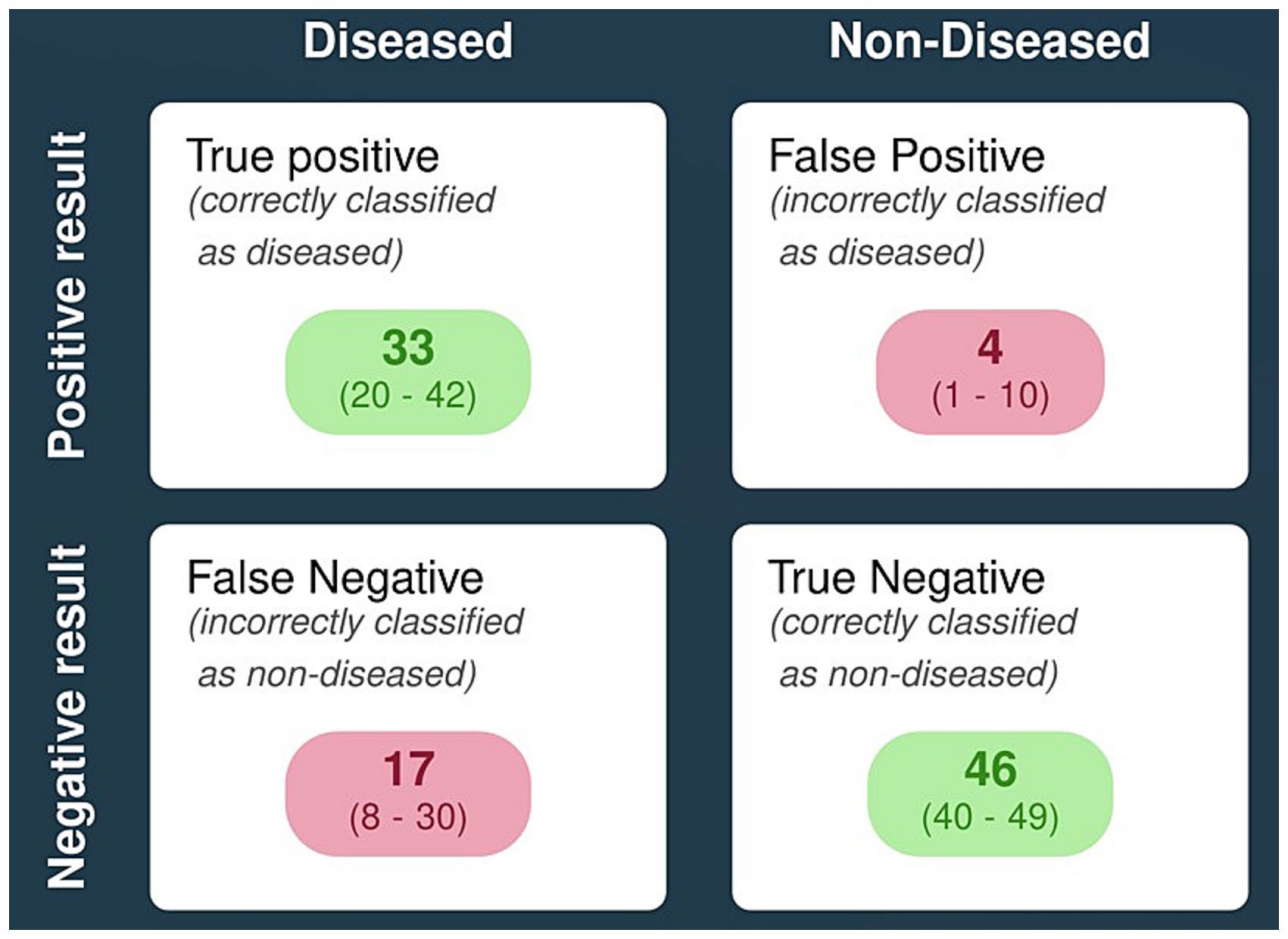
Pictorial representation of diagnostic performance of LAMP for detecting scrub typhus using a hypothetical population model. [Note: proxy prevalence of scrub typhus is taken to be ~50%; *n* = 100].

Figure 5 represents a hypothetical scenario wherein a population of 100 with 50% disease prevalence accurately depicts the diagnostic performance of LAMP as an isothermal assay for the detection of scrub typhus.

## 4 Discussion

Rickettsial infection is one of the most significant zoonosis around the globe and continues to threaten the lives of millions. Its diagnoses pose a major challenge to clinicians and researchers; commercial diagnostic assays readily available at tertiary healthcare settings are otherwise indispensable at resource poor, point-of-care settings. In the pursuit of this research gap, the current study aimed to explore, estimate, and quantify the performance of isothermal assays, optimized to detect and diagnose classical rickettsial diseases. Overall, six studies were selected as a part of this systematic review that employed LAMP as their primal index test for the detection of scrub typhus (STG). All the studies were assessed for their quality and their potential risk of bias; a narrative synthesis approach was employed to comprehend and compare the descriptive findings. A meta-analysis was pursued to assess and quantify pooled effect estimates across studies to determine the diagnostic performance of the index test. The results from the meta-analysis suggest existence of severe heterogeneity between the studies, and so, a meta-regression was performed to explain its origins. Significance of results in the light of existing literature, quality concerns, general comments, limitations, and the future directions of the current study are discussed herewith in this section.

Eliminating the effects of outlier values (low sample size) in the sensitivity analysis, the results from the study report pooled sensitivity of LAMP to be 0.59 [95% CI; 0.31–0.82] and specificity to be 0.92 [95% CI (0.77–0.97)]. However, in the bivariate analysis, the value rises up to 66% [95% CI (0.40–0.85)] and 94% [95% CI (0.81–0.98)] for both sensitivity and specificity, respectively. A similar and an interesting exaggerating effect can be spotted in the meta-regression analysis which was pursued to explore potential heterogeneity encountered in this study. The usage of a case–control study design in diagnostic accuracy studies may lead to exaggeration of sensitivity and specificity, coherent with prior literary evidence (Aliu and Chung, 2012; Lijmer et al., 1999). The process of selection of cases and controls itself serves as an over-estimation of the diagnostic indicators; the sampling technique employed in case–control study often leads to selection of subjects that either present as extreme (cases) or no symptoms at all (controls). This creates an artificial bifurcation that can influence the test to perform better than it would in the real world (mixed population setting with an observational/cross-sectional study design, where cases occur more natural in different spectrums). Using a pre-determined sample size in case–control studies can additionally cause extrapolation of the predictive diagnostic indicators (positive predictive and negative predictive value) that tend to be influenced by endemicity and diseases prevalence. Moreover, subclinical or borderline cases that may not appear diseased are often overlooked in case–control settings, in turn causing the over-estimation with defined cases and controls since they are more likely to produce a positive or negative result (making the diagnostic performance appear to be better). Finally, a variation in the chosen diagnostic assay may also influence the assignment of cases and controls; a gold-standard assay will be the best possible diagnosis; however, it may not be feasible to implement, leading to usage of alternative assay, a potential verification bias.

Parallelly, a cross-sectional study design may provide better estimation and exert lesser influence which may be a cause of these exaggerations. The results, although not significant, reflect important inferences and provide unique statistical evidence that supports the influence of a case–control study design on a diagnostic accuracy study. The STARD guidelines (Cohen et al., 2016) that explain a step-by-step protocol and reporting standards on the clinical and statistical validation of diagnostic accuracy studies, vouch for the importance of a similar argument and thus discourage the use of case–control designs. In addition, two of the selected studies opted to use a case–control design (Kannan et al., 2020; Anitharaj et al., 2023), which led to a potential risk of bias and quality concerns in each of these studies in accordance with the QUADAS-2 scoring system.

Due to stringency in the inclusion and exclusion criteria, a significant number of studies from the SFG and TG were disqualified during the screening phase. This was evidently done to avoid inclusion of studies that do not completely adhere to STARD guidelines. Resource limited settings and restricted funds are the primary reasons for choosing commercial diagnostic assays as reference tests that may lead to improper validation and insignificant results. Some of the excluded studies opted for PCR (Gui et al., 2022; Carvajal-Gamez et al., 2024; Raele et al., 2018), qPCR (Kim et al., 2018), and culture-based isolation (Dittrich et al., 2014) as their gold standards, when IFA has been the choice of reference test for diseases caused by *Rickettsia* species (Stewart and Stewart, 2021). Interestingly, IFA is not a foolproof diagnostic assay, as the use of arbitrary cutoff without establishing titers in the healthy local population has been raised multiple times as a major weakness (Kala et al., 2020; Blacksell et al., 2007; Vanlalruati et al., 2022), in addition to the its processing cost, expensive set-up, and requirement of trained manpower. PCR and ELISA, although not being gold standards, have been routinely used for the detection of *Rickettsia* and provide an economical and reliable alternative for clinicians and researchers (Abdad et al., 2018; Stewart and Stewart, 2021; Luce-Fedrow et al., 2015). Nonetheless, IgM ELISA poses its own limitations as the cutoff for anti-OT IgM by ELISA varies by geographical location depending on zonal endemicity; thus, a standardized cutoff value is often unavailable (Jain et al., 2023). A cost-effective, high-end performing and dispensable diagnostic test, preferably isothermal in nature, is an urgent and imperative necessity for timely and accurate diagnosis of *Orientia* species.

The systematic review and meta-analysis described in this study originally attempted to quantify the diagnostic performances of isothermal assays in the detection of classical rickettsial diseases belonging to three major groups: SFG, STG, and TG. However, during the screening and selection, none of the studies belonging to the SFG and TG could be included in the review due to stringency of the inclusion and exclusion criteria. Thus, during the peer review process, referees had suggested to modify the title of the review to “*The diagnostic accuracy of point-of-care (PoC) nucleic acid-based isothermal amplification assays for scrub typhus: A systematic review and meta-analysis*.” This was done to correctly index the article under the appropriate category and ensure the scope of the article is accurately projected. The Prospero protocol of the given review is currently indexed under the original title “*The diagnostic accuracy of Point-of-Care (PoC) nucleic acid-based isothermal amplification assays for scrub typhus: A systematic review and meta-analysis*.”

The study although contributes to significant findings in the field of scrub typhus diagnostics is, however, met by certain limitations. During the screening and selection phases, bibliometric mining was adapted as an additional search strategy to enhance inclusivity of reports. The bibliometric mining was performed in limited studies by only one reviewer in reports accessible in full text, which could be a potential source of bias. Moreover, during the meta-analysis, certain covariates were selected to explore the causes of heterogeneity in between the studies. There might be existence of other covariates that remain unexplored and may have contributed to the extensive heterogeneity encountered in between the studies. Finally, selection and screening of the studies was solely based on STICS criteria for scrub typhus group; however, a modified scrub typhus infectious disease criteria adapted by Blacksell et al. (2018) provides a precise, highly sensitive, and specific criteria for the detection and screening of scrub typhus. This could serve as a potential minor concern in terms of selection and screening of studies using the STICS criteria, employed in the given study.

## 5 Conclusion

The systematic review and meta-analysis described in this study provides significant and conclusive findings supported by statistical evidence that vouch for optimization of novel isothermal assay for detection of rickettsial diseases. Although LAMP, trusted for its optimum diagnostic performance, may not be an ideal choice of an isothermal assay for the detection of scrub typhus. This calls for active research and development, dedicated toward optimization of novel diagnostic techniques that may serve to screen pathogenic agents such as *Rickettsia* and *Orientia*, in a timely manner. Synergy between various stakeholders (researchers, physicians, industry, and policymakers) is required to develop and deploy diagnostic assays that fit in paradigm of Affordable, Sensitive, Specific, User-friendly, Rapid and robust, Equipment-free and Deliverable to end-users (ASSURED) criteria set by WHO, so as to bridge the gap between delayed diagnosis and treatment.

### Additional information

Items reported in this systematic review (protocol, flowchart and guidelines) were followed in accordance with PRISMA 2020. All the templates and reporting standards can be accessed at http://www.prisma-statement.org/PRISMAStatement/. Risk of Bias and quality assessment were performed using the QUADAS-2 tool. A detailed guide, signaling questions and toolkit is available at https://www.bristol.ac.uk/population-health-sciences/projects/quadas/quadas-2/. A detailed protocol pertaining to this systematic review is registered (CRD42024511706) and can be accessed at https://www.crd.york.ac.uk/prospero/.

## Data Availability

The original contributions presented in the study are included in the article/Supplementary material, further inquiries can be directed to the corresponding author/s.

## Footnotes

^1^ https://ciberisciii.shinyapps.io/MetaDiSc2/
