# Supplementary material for: The diagnostic accuracy of point-of-care nucleic acid-based isothermal amplification assays for scrub typhus: a systematic review and meta-analysis
Source: Front Microbiol. 2025 Jan 6;15:1516921. doi: 10.3389/fmicb.2024.1516921 (PMC11743491; doi:10.3389/fmicb.2024.1516921)
Supplement: Supplementary file 1 [file Table_1.docx]

**Online Supplement to: “**The diagnostic accuracy of point-of-care (PoC) nucleic acid-based isothermal amplification assays for scrub typhus: A systematic review and meta-analysis.”

**Supplementary file 1**

**Table A:** Diagnostic parameters as reported in each study required to generate a 2 by 2 contingency table for estimation of epidemiological and diagnostic indicators. (Key: TP- true positive, FP-false positive, FN- false negative, TN- true negative).

| **Study** | **a (TP)** | **b (FP)** | **c (FN)** | **d (TN)** |
| --- | --- | --- | --- | --- |
| Anitharaj, V. et al 2023 | 102 | 42 | 38 | 98 |
| Paris et al 2011 | 29 | 6 | 26 | 100 |
| Kathikeyan et al 2019 | 33 | 0 | 5 | 13 |
| Roy et al 2021 | 6 | 2 | 31 | 235 |
| Kannan et al 2020 | 145 | 36 | 13 | 122 |
| Blacksell et al 2012 | 29 | 6 | 25 | 100 |

**Table B:**  Basic summary statistics of the meta-analysis.

| **Coefficient** | **Estimate** |
| --- | --- |
| Studies | 6 |
| Number of diseased | 482 |
| Number of non-diseased | 760 |
| Total | 1242 |
| Prevalence | 0.39 |
|  |  |

**Table C:** A glimpse of summary statistics and heterogeneity analysis in the Univariate model of the meta-analysis.

| **Summary stats** | | | | **Heterogeneity Analysis** | |
| --- | --- | --- | --- | --- | --- |
|  | Estimate | 95% LCI | 95% UCI |  | Estimate |
| Sensitivity | 0.661 | 0.404 | 0.849 | Var logit(sen) | 1.642 |
| Specificity | 0.935 | 0.811 | 0.98 | Var logit(spe) | 1.877 |
| DOR | 28.178 | 5.635 | 140.894 | MOR sensitivity | 3.395 |
| LR+ | 10.214 | 3.108 | 33.561 | MOR specificity | 3.695 |
| LR- | 0.362 | 0.179 | 0.733 | I2 sensitivity | 0.937 |
| FPR | 0.065 | 0.02 | 0.189 | I2 specificity | 0.909 |
